# Supplementary material for: Metabolic modelling reveals the aging-associated decline of host–microbiome metabolic interactions in mice
Source: Nat Microbiol. 2025 Mar 26;10(4):973–91. doi: 10.1038/s41564-025-01959-z (PMC11964932; doi:10.1038/s41564-025-01959-z)
Supplement: Supplementary file 1 — Supplementary results, methods and references. [file 41564_2025_1959_MOESM1_ESM.pdf]

nature microbiology

Supplementary information https://doi.org/10.1038/s41564-025-01959-z

Metabolic modelling reveals the aging-associated decline of host–microbiome metabolic interactions in mice

In the format provided by the authors and unedited

Supplementary Results

Age-specific simulation of drop-out communities

We investigated the relevance of aging-associated microbial species for microbial community metabolism. To this end, we repeated community simulations of microbiomes from the mouse cohort while individually dropping aging-regulated strains from the microbiomes of old or young mice. Overall, community growth was impacted negatively when species that go down with age were removed, while the community growth increased upon deletion of aging-induced MAGs (Extended Data Fig. 4D-E). Thus, MAGs with reduced abundance during aging tend to have a positive impact on community productivity and MAGs that increase in abundance with age tend to have a negative impact. Overall, the removal of species had contrasting effects depending on community age. In communities of young mice (2 months), species removal generally led to a decline in community productivity, suggesting a high degree of interdependence among species. In contrast, species removal in communities of older mice (24 and 30 months) typically resulted in increased community growth rates, indicating that competitive interactions predominated in these communities.

Analysis of shifts in microbial ecological strategies

We investigated the extent to which aging-associated shifts in microbiome ecology were related to species-level ecological strategies according to the universal adaptive strategies theory (UAST) framework, which categorizes species into ruderals, competitors, and stress tolerators [37,38]. Within this framework, ruderals are species that are first colonizers of niches characterized by rapid growth and low catabolic diversity, competitors are species that can outcompete other species through direct antimicrobial effectors and broader resource utilization, and stress tolerators are species that are resistant to stress. We observed a significant increase in the ruderal strategy with age, whereas stress tolerators and competitors significantly decreased in frequency (Extended Data Fig. 4F). While having high growth potential, the reduced catabolic diversity of ruderals and their focus on de novo colonization of niches makes them poor interaction partners in metabolic cross-feeding interaction, thereby reducing the overall capacity of the community to grow due to less efficient utilization of dietary resources.

Conserved aging-associated changes across mouse tissues

We conducted a differential gene expression analysis followed by GO enrichment of the significantly changed transcripts to gain insights into aging-associated changes in the host. Consistent with our previous observation of a signature of aging conserved across species and tissues [3], we observed a strong conserved signature of aging across the colon, liver, and brain in our mice. Overall, we observed increased activity for inflammatory and immune processes in aged mice across all three tissues (inflammaging) and decreased proliferative potential in the colon and brain. Other examples of aging-induced processes shared across all tissues were lipopolysaccharide (LPS), defense, immune, and inflammatory responses, along with the formation of blood vessels (Fig. 5A). Nervous system development was downregulated in both

the aged colon and brain, which appeared to occur at an earlier age in the colon (9–15 months) than in the brain (15–24 months). The downregulation of cell division was unique to the aged colon (Extended Data Fig. 5B). In the liver, processes related to mitochondrial energy production, protein translation, and assembly showed age-related decreases, whereas immune processes, signaling pathways, and proliferation processes (such as cell migration, cell proliferation, and extracellular signal-regulated kinase cascades) showed age-related increases (Extended Data Fig. 5C). In the brain, gene expression related to microglial cell activation and LPS-related and pattern recognition signaling pathways showed age-related increases, whereas many learning, memory, synaptic plasticity, and synaptic signaling pathways showed age-related decreases (Fig. 5A, Extended Data Fig. 5D). Focusing on gene-level conservation of aging-associated regulation, we found 157 transcripts that were consistently downregulated with age and 526 transcripts that were consistently upregulated with age. The significantly induced genes were enriched for regulation of interleukin (IL)-1b, IL-2, IL-4, IL-6, IL-10, IL-12, IL-17, tumor necrosis factor (TNF), interferon (IFN)-α, IFN-β, IFN-ɣ, C-X-C motif chemokine ligand 2 (CXCL2), and immunoglobulin and cytokine production (Extended Data Fig. 5A; Supplementary Tables S5.7–S5.9). Because we found an aging-associated increase in microbial production of the pro-inflammatory microbial metabolite succinate [36,54] (Fig. 4C), we further explored how host genes, associated with microbial succinate metabolism, were regulated during aging. In the colon, we found bacterial interconversion of oxalate to succinate in the oxalate degradation pathway negatively associated with age and positively with host DNA repair, cell division and cell adhesion. Moreover, a bacterial succinate to succinyl-CoA interconversion reaction of the itaconate degradation pathway was positively associated with host age and negatively associated with host side protein processing (catabolism, phosphorylation, ubiquitination) as well as telomere maintenance and DNA damage response (Fig. 6C, Supplemental Table S2.1). This indicates that besides a pro-inflammatory role, aging-associated changes in microbial succinate production could potentially contribute to a reduced DNA damage response as one of the hallmarks of aging [2,55].

Supplementary Methods

Comparing metabolic models via principal component analysis

Metabolic models were used to create an incidence matrix of all available reactions per model. The incidence matrix was normalized sample wise in order to weigh the importance of reactions by the size of the model. This means larger models with more reactions had lower weight per reaction compared to smaller models containing less reactions. Dissimilarity between bacterial metabolic models was calculated from this incidence matrix via Horn–Morisita index as implemented in the function vegdist from R-package vegan v2.6-4. Principal Component Analysis was performed on these dissimilarity indices using the function prcomp from the R-package stats v4.3.2. Metadata information of each model, namely count of tRNAs, completeness, contamination, GC-content, genome size, model size and model gaps, was fitted to the principal coordinates with the function envfit (vegan v2.6-4). The first two principal components were plotted together with the metadata vectors in Fig. 1B. Explained variance for

model fitting of each metadata variable separately against the first two principal components was reported as R^2 values in the text.

Estimation of functional capacity of microbiomes

Flux variability analysis (FVA) was used to calculate all possible flux ranges for each reaction in each metabolic model constrained by the ssniff diet. The biomass production was set as the objective function to obtain flux ranges that satisfied maximal model growth (99% percent of maximal growth used as cut-off). A binary incidence matrix was constructed with bacteria as columns and biochemical reactions as rows. All reactions that carried a non-zero ($>10^{-6}$) absolute flux were assigned "1". This matrix was first normalized to the number of active reactions in each bacterium and then multiplied by each mouse's bacterial abundances to obtain a reaction abundance for the microbiome of each mouse. Finally, the reaction abundance table was normalized by the sum of each mouse's reaction scores, making the resulting relative reaction abundances comparable between mice.

Inferring microbial niche strategies via UAST

Microbial life history traits, defined by the universal adaptive strategies theory (UAST) framework [37,107], were predicted (similar to a previous study [38]). Microbial traits were inferred by gapseq (version 1.2; sequence DB md5sum: bf8ba98) [24] using MetaCyc pathways [26], Bakta v1.8 [108], abricate (version 1.0; https://github.com/tseemann/abricate) with the virulence factor database [109], and gRodon (version 2.3) [110]. The competitive strategy was defined by the following traits: genome length (high quantities), antibiotic biosynthesis pathways (high), siderophore biosynthesis pathways (high), and catabolic pathways (high). The stress toleration strategy was defined by rRNA genes (low quantities), biofilm genes (high quantities), and auxotrophies (high quantities). The ruderal strategy was defined by catabolic pathways (low quantities), rRNA genes (high quantities), and codon usage bias (high quantities). Given the distribution of each trait among all species, a species' trait belonging to the 0.75 (traits with high quantities) or 0.25 (traits with low quantities) quantile was considered to contribute to a strategy. The number of contributing traits was summed for each strategy, and the strategy with the highest number of contributing traits was considered a species' final life history strategy. In the case of multiple strategies with the same highest number of contributing traits, multiple strategies were assumed to be relevant. In addition to the 0.75 and 0.25 quartiles, different percentiles (0.7/0.3 and 0.8/0.2) were tested to assess stability.

The niche strategies of all species from one microbial community (per mouse) were summed, with a down-weighting of the impact of species with two equally likely strategies by 0.5 and a down-weighting of the impact of species with three equally likely strategies by 0.333. The niche strategy abundances were normalized by sample and multiplied by 100 to obtain percentages. Differences between age groups were tested for each of the three possible niche strategies using Kruskal–Wallis tests with pairwise post hoc Dunn's tests and Benjamini–Hochberg FDR correction (using the DunnTest function of the R DescTools package [version 0.99.50]) and plotted (Supplementary Fig. 4F).

Microbiome community modeling

The microbial communities were modeled using FBA to study the association between the microbiome metabolic network and the age of the mouse hosts. FBA is a mathematical approach for studying metabolic networks built from all known reactions in an organism. By estimating the flow of metabolites in the network, FBA allows the prediction of the growth rate of the organism and the fluxes of all metabolites. This is carried out under the evolutionary assumption that the preferable path maximizes the biomass compounds. For prediction of microbial community fluxes, we used community FBA [17], a variant of FBA working on the community level. To this end, the metabolic networks of different microbial species within the community were connected in a common compartment for metabolic exchange within the community and with the environment (the host's intestinal tract). A community-level biomass reaction was introduced that consumed the biomass of individual species according to their relative abundance as inferred from metagenomic data. Additionally, we introduced coupling constraints to prevent excessive flux through individual microbes' reactions' without concomitant growth (coupling parameters c=400, u=0.01). For predicting maximal growth, we optimized the community-level biomass reaction while subtracting the total sum of fluxes across all reactions multiplied with a factor of 10^{-6} to obtain a parsimonious solution. No feasible solution could be obtained for two microbiome communities from age group 30, leaving 50 communities for downstream analysis (10 for each age group). The constant outflow of bacterial biomass via feces requires a constant growth of resident bacterial species to maintain population density and therefore supports optimization of growth as a reasonable assumption for modeling gut microbial communities. Also, our community modeling approach assumes a community-level optimization of community growth rates which might not be realistic in all scenarios as it assumes a coordination of metabolic fluxes between species to maximize overall growth but comes at the advantage of a considerable speed up in computational time compared to approaches that consider only an individual-level optimization of growth rates such as, e.g. individual-based modeling approaches [111].

This analysis identified three types of reaction fluxes. Exchange fluxes of metabolites exported and taken up from outside the community were considered metabolites that may be exchanged with the host (Fig. 4C). Metabolites shared between different microbes were represented by reaction fluxes that were exchanged among members of the microbial community (Supplementary Fig. 4B). Finally, the reaction fluxes within each respective bacterial model were considered as internal reaction fluxes (Fig. 4B). Additionally, this analysis yielded a predicted community growth rate which, when multiplied with the individual species' abundances, allows to derive the growth rate of each individual bacterial species (Fig. 4D).

All three types of reaction fluxes were normalized to the community growth rate and analyzed separately. Absolute flux values were correlated with age using Spearman's rank correlation coefficient (using the cor.test function in the R package stats [version 4.3.2]) separately for each reaction flux. The *p*-values were corrected for multiple testing with the Benjamini–Hochberg FDR method, and only results with FDR-adjusted *p*-values of ≤0.05 were plotted (Fig. 4B, C, and Supplementary Fig. 4B).

Significant age-correlated internal reaction fluxes were enriched for MetaCyc pathways, stratified by positive and negative correlations, with an overrepresentation test implemented in the enricher function of the R package clusterProfiler (version 4.8.3). The pathways with an

enrichment *p*-value of ≤0.05 and at least three enriched features were reported (Fig. 4B; Supplementary Tables S4.2–S4.4).

Microbiome community modeling with single microbe deletion

In order to evaluate the impact of single microbiome members on community interactions and growth we ran several microbiome community modeling instances where we selectively deleted a single member. To this end we choose the 20 MAGs with their abundance most significantly changing during aging (10 MAGs going up with age and 10 MAGs going down with age, Fig. 4A) for deletion from 50 microbial communities (n = 10 per age group). We implemented this deletion approach by removing only one model (MAG) at a time from each individual mouse microbiome community and simulated metabolite exchange as well as community growth via FBA as described above ("Microbiome Community Modeling"). We obtained the change in community growth rates by subtracting the new growth rate of a community lacking one member from the original community growth rate (Supplementary Table S4.9). First, we looked into the change of growth in all 50 communities stratified by the MAG that was removed and observed a decrease in community growth upon removal of 8 out of the 10 aging down-regulated MAGs, while 2 did not impact community growth. Removal of aging up-regulated MAGs led, in 9 out of 10 cases, to increased community growth rates (Supplementary Fig. 4D). In a second analysis we compared the change of community growth stratified by deletion of aging down- or up-regulated MAGs and by age-group of the host (Supplementary Fig. 4E). We performed Wilcoxon's rank sum test between deletion of down- or up-regulated MAGs within each age-group and corrected for multiple testing via Benjamini and Hochberg's method. We observed a significant increase of community growth rates upon deletion of up-regulated MAGs starting from age 9 months and an increased impact of these MAG-deletions on community growth with older host age.

Transcriptome sequencing

Main study cohort

RNA was reverse-transcribed into Illumina shotgun sequencing libraries with TruSeq RNA stranded kit and polyA enrichment following the manufacturer's protocol (Illumina, San Diego, CA) at the Competence Centre for Genomic Analysis (Kiel, Germany) and sequenced for 2 × 75 cycles (2 × 100 for colon) in paired-end mode with ~13 samples per lane on an Illumina HiSeq4000 machine. Demultiplexing was conducted with zero mismatches allowed in the barcodes. Illumina TruSeq adapter sequences were trimmed from the forward and reverse reads with Cutadapt (version 1.12) with a minimum sequence overlap of 3 bp and no more than 10% mismatches; they were also filtered for a minimum read length of 20 bp and trimmed for 3'-end quality using a Phred score of ≥30. Additional quality filters were applied with Prinseq lite (version 0.20.4), allowing at most eight unknown nucleotides ("N") per read and requiring an overall mean Phred score (read quality) of ≥15; the reads were also trimmed for 5'-end quality using a Phred score of ≥12.

The filtered reads were mapped against the *Mus musculus* reference genome (GRCm38.99) in Hisat2 (version 2.1.0) with the RNA strandedness set to FR, applying non-deterministic random seeds and suppressing mixed alignments of read pairs. Only primary alignments for each read were kept, via a filtering step (-F 256) in Samtools (version 1.9). Gene counts were extracted in HTSeq count (version 0.6.1) with reverse-stranded information in union mode.

Germ-free mice cohort

RNA was reverse-transcribed into Illumina shotgun sequencing libraries with TruSeq RNA stranded kit and polyA enrichment following the manufacturer's protocol (Illumina, San Diego, CA) at the Competence Centre for Genomic Analysis (Kiel, Germany) and sequenced for 2×100 cycles in paired-end mode on an Illumina NovaSeq6000 machine. Demultiplexing was conducted with zero mismatches allowed in the barcodes. Reads were mapped using the nextflow pipeline of nf-core/rnaseq (https://nf-co.re/rnaseq). In short, the reads were quality trimmed using trimGalore (version 0.6.7) with a minimum phred score cutoff of 20 and a minimum read length of 20 bp and potential PCR-duplicates were removed with dupRadar (version 3.14) using defaults. For quality control we employed fastQC (version 0.11.9) and multiQC (version 1.10.1). Quality processed reads were mapped against the GRCm39 mouse reference genome using STAR (version 2.6.1d) and read counts were estimated using salmon (version 1.5.2) with default parameters.

Differential gene expression analysis

Main study cohort

Differentially expressed genes were identified using the R package DESeq2 (version 1.40.2) [91]. The samples were stratified by organ (colon, liver, and brain) and then analyzed with a design formula that accounted for the age (numeric, centered, and scaled) and the sequencing batch, if applicable (liver and brain). Genes differentially expressed according to age were reported, controlling for independent filtering at 0.05 using DESeq2 (function: results, variable: alpha). Genes with an adjusted *p*-value of ≤0.05 were considered significantly differentially expressed. Differentially expressed genes were stratified by their positive or negative association with age and annotated separately with GO biological processes via enrichment analysis using the enricher function of the R clusterProfiler package (version 4.8.3). For plotting (Fig. 5), the GO terms were filtered using an FDR-adjusted *p*-value cutoff of 10^{-4} for the colon and 10^{-6} for the liver and brain; redundant higher-level GO terms were then removed, via the filtering algorithm described in [112].
Variance-stabilizing transformed gene abundance data were exported for downstream use with host–microbiome partial correlations using DESeq2 (function "getVarianceStabilizedData" with parameter blind = FALSE). For the reconstruction of the metamodel, all samples were stratified by organ and processed with a design formula accounting for the sequencing batch and age group. The results were extracted from the DESeq results object for each of the 10 possible pairwise age group comparisons.

Germ-free mice cohort

Differentially expressed genes were identified using DESeq2 (version 1.42.0). Samples were stratified by the five tissues and analyzed using a design formula that set treatment group as the primary variable. For each organ, DESeq2 datasets were created and re-leveled to use GF as a reference level compared to CONVD or CONVR, ensuring that comparisons are made against the appropriate baseline condition. This approach allows the detection of gene expression changes specifically attributable to differences in microbiome status. Contrasts were defined to compare conditions of interest (i.e., CONVD vs. GF, CONVR vs. GF) capturing differences in gene expression driven by microbial colonization.

Functional and taxonomic profiling of metagenomic reads

We employed the Biobakery3 pipeline [27] to infer functional (HUMAnN3.6 with database "v201901_v31") and taxonomic associations (MetaPhlan4.0.4 with marker gene database "mpa_vJan21_CHOCOPhlAnSGB_202103") directly from quality controlled metagenomic reads. Mapping rates against the biobakery databases was low with an average alignment rate of 41.5 % (range: 31.3 - 67.5 %), as compared to an average alignment rate of 89.7 % (range: 81.5 % - 96.7 %) when mapping against our MAGs. The taxonomic annotations were filtered to species level and checked for changes in species abundance with host age via linear models (Supplementary Fig. 4A) comparable to the approach described in the methods section "MAG assembly and annotation". Due to different taxonomic databases and methods of annotation the resulting species names might differ from those of our MAG-based analysis. The functional predictions were regrouped from genefamilies to MetaCyc reactions with humann3 utility scripts and then used for colon transcriptome to microbiome reactions partial correlations (Supplementary Fig. 2B) as described in the methods section "Host-Microbiome partial correlations". Instead of subsystems for higher-level grouping and reaction annotation, MetaCyc pathways were used here.

Non-targeted metabolomics using HILIC UHPLC-MS/MS

Fecal pellets (~40 mg) were weighed in sterile ceramic bead tubes (NucleoSpin Bead Tubes; Macherey-Nagel, Dueren, Germany) and extracted with 1 mL of chilled methanol (−20 °C; LiChrosolv, Supelco; Merck KGaA, Darmstadt, Germany). Fecal matter was homogenized and extracted with a Precellys Evolution Homogenizer (Bertin Corp., Rockville, MD, USA; 4,500 rpm, three 40-second cycles with a two-second pause). The samples were centrifuged for 10 minutes at 21,000 ×g and 4°C, and the supernatant was transferred into sterile tubes until analysis. Next, 100 µL of fecal methanolic extract was evaporated at 40°C with a SpeedVac concentrator (Savant SPD121P; Thermo Fisher Scientific, Waltham, MA, USA) and reconstituted in 75% acetonitrile (ACN; LiChrosolv, hypergrade for LC–MS; Merck KGaA), spiked with L-leucine-5,5,5-d_3 at 5 mg/L (99 atom % D; Merck KGaA), which was prepared in a methanol and water solution (50:50).

The samples were analyzed by using a UHPLC system (Acquity; Waters, Eschborn, Germany) coupled to a quadrupole time-of-flight (TOF) mass spectrometer (maXis; Bruker Daltonics, Bremen, Germany), as described previously [113]. Mass spectra were acquired at electrospray ionization of positive and negative modes (+/−). Spectrometric data were acquired in line and

profile mode with an acquisition rate of 5 Hz from 50 to 1500 Da. Fragmentation experiments were set to the data-dependent mode (MS/MS [Auto]), where the three most intense ions were fragmented within one scan when a count reached over 2000. Ions were excluded after acquiring three MS/MS and reconsidered for fragmentation after six seconds. The collision energy was set to 20 eV for both modes with an isolation width of 8 Da. The electrospray ionization source parameters were as follows: capillary voltage of 4500 V for (+) and 4000 V for (−), end plate offset of (+/−) 500V, nebulizer gas of 2 bar, dry gas of 10 L/min, and dry heater of 200°C. Before measurements, the MS was calibrated using the ESI-L Low Concentration Tuning Mix (Agilent, Santa Clara, CA, USA). The ESI-L Low Concentration Tuning Mix (diluted 1:4 [v/v] with 75% ACN) was injected in the first 0.3 min of each run by a switching valve for internal recalibration by post-processing software.

Ammonium acetate (NH_4Ac; LiChropur eluent additive for LC–MS; Merck KGaA) at 0.5 mol/L was adjusted to pH 4.6 with glacial acetic acid (Honeywell; Fluka, Seelze, Germany). Milli-Q water was obtained from a Milli-Q Integral Water Purification System (Billerica, MA, USA). Polar metabolites were separated by HILIC by using an iHILIC-Fusion UHPLC column SS (100 × 2.1 mm, 1.8 µm, 100 Å; HILICON AB, Umea, Sweden). The eluent compositions were as follows: Eluent A consisted of 5 mmol/L NH_4Ac (pH 4.6) in 95% ACN (pH 4.6), and eluent B consisted of 25 mmol/L NH_4Ac (pH 4.6) in 30% ACN. We started with 0.1% B, keeping it constant for two minutes, then increased B to 99.9% over 7.5 minutes. The condition of 99.9% B was kept for two minutes and reversed to 0.1% B within 0.1 minutes, held for 0.1 minutes. The run was completed after 12.1 minutes, and the column was equilibrated for five minutes before the next injection. The flow rate was set to 0.5 mL/min, the column temperature to 40°C, and the sample manager was cooled to 4°C; 5 µL of the sample was injected into the column (partial loop). The weak and strong washes consisted of 95% and 10% ACN, respectively.

Metabolite identification and metabolomic data processing

The raw LC–MS data were post-processed in GeneData Expressionist Refiner MS (version 13.5.4; GeneData GmbH, Basel, Switzerland), including chemical noise subtraction, internal calibration, chromatographic peak picking, chromatogram isotope clustering, valid feature filter (cut-off of 100 [+] or 2000 [−] maximum intensity and presence of features in at least 20% of samples for [+/−]), retention time range restriction (0.4–10.7 minutes), annotation of known peaks (mass-to-charge tolerance of 0.01–0.005 Da and retention time tolerance of 0.1), and MS/MS consolidation and export to merged MASCOT generic files (MGFs). Data processing resulted in a matrix containing features with mass-to-charge ratios (*m/z*), retention times, and observed maximum intensities for each sample. The data were normalized to the weighed-in wet fecal weight and a maximum intensity of 5 mg/L L-leucine-5,5,5-d_3.

The merged MGF files were used to search a spectral library using MSPepSearch (0.01 Da mass tolerance for precursor and fragment searches). Experimental and *in silico* spectral libraries were downloaded from MassBank of North America (https://mona.fiehnlab.ucdavis.edu/). Identification was performed by matching experimental MS/MS spectra against MS/MS of spectral libraries downloaded from MassBank of North America using MS PepSearch (release: 02/22/2019; 0.01 Da mass tolerance for precursor and fragment searches). From the MS PepSearch output, features with the highest dot product of the same identifier were retained, and then metabolites with a dot product of <500 were removed. Furthermore, Global Natural Products Social (GNPS) Molecular Networking and Library Search were used to identify metabolites in the experimental MS/MS data [114]. Therefore, for each feature from the metabolite data table, an MS/MS was selected based on the highest total ion count. The MS/MS with the highest count was submitted to GNPS (201 features for [+]

and 361 features for [−]). The GNPS Library Search was conducted with the following settings: The precursor ion mass tolerance and fragment ion mass tolerance were set to 0.01 Da, minimum matched peaks were set to 1, and the score threshold was set to 0.5. GNPS Molecular Networking was performed with the following settings: the precursor ion mass tolerance was set to 0.01 Da, the MS/MS fragment ion tolerance was set to 0.01 Da, and the cosine score was set to >0.5 with a minimum matched peak of 1, TopK was set to 10, the maximum component size was set to 100, the maximum shift was set to 200 Da, the minimum cluster size was set to 1, and the maximum analog search mass difference was set to 500. Bile acids were also annotated in the (−)) HILIC dataset with an error window of 0.005 Da, taking the following adducts into account: $[M\text{-}H]^-$, $[M+Cl]^-$, $[2M\text{-}H]^-$, and $[M+CH_3CHOO]^-$. Seven bile acids conjugated with sulfate were putatively annotated as $[M\text{-}H]^-$ with an error window of 0.005 Da, resulting in 53 features. To confirm the identity of bile acids, we performed an identification step with semi-targeted peak picking of bile acids, as described by Sillner et al. [115], with a UHPLC system (ExionLC; AB Sciex LLC, Framingham, MA, USA) coupled to a quadrupole TOF mass spectrometer (X500 QTOF MS; AB Sciex LLC). The chromatographic settings were identical to those used by Sillner et al., whereas MS detection was performed by the Q-TOF instrument in (−) ionization mode (TurboIonSpray; AB Sciex LLC). The curtain gas was set to 30 psi, ion source gases 1 and 2 were set to 45 psi, and the temperature was set to 500 °C. The MS was operated in the TOF MS and TOF MS/MS scan mode. The TOF MS analyzed molecules between 65 and 1000 Da. The ion spray voltage was set to −4500 V, CAD gas was set to 7, accumulation time was set to 0.1 seconds, the declustering potential was set to −50 V, the declustering potential spread was set to 0 V, the collision energy was set to −5 V, and the collision energy spread was set to 0 V in TOF MS mode. In TOF MS/MS mode, information-dependent acquisition (small molecule) was selected, and the following parameters were used: a maximum of 10 candidate ions, an intensity threshold of 1000 counts per second, activated dynamic background subtraction, excluding isotopes (±4 Da), and mass tolerance of ±50 mDa. The TOF MS/MS acquired data between 50 and 1000 Da, with an accumulation time of 0.025 seconds, a declustering potential of −80 V, a declustering potential spread of 0 V, a collision energy of −35 V, and a collision energy spread of 15 V; the Q1 resolution was set to unit.

Forty-five bile acids (0.05–0.4 mg/mL in methanol) and 50 µL of fecal methanolic extracts of 24 mice (two [n = 5], 9 [n = 5], 15 [n = 4], 24 [n = 5], and 30 [n = 5] months) were analyzed for bile acid identification. Bile acid retention times (0.05 mg/mL) were manually selected using Sciex OS Analytics (version 3.0.0.3399) with an extraction width of 0.02 Da. Raw files (.wiff2) derived from the LC–MS-based separation of bile acids were processed as described above, including chemical noise subtraction, chromatographic peak picking, chromatogram isotope clustering, and annotation of known peak function (with an rt tolerance window of 0.1 minutes and an m/z tolerance of 0.005 Da). The bile acid data (m/z, rt, and maximum intensity values for each sample) were normalized to the wet fecal weight. Pearson's correlation was performed between the bile acid data and the (−) HILIC data subset (annotated bile acids), and features with coefficients >0.8 were considered for manual identification. Using this approach, 47 features were identified, including different adducts, and 23 features were identified as $[M\text{-}H]^-$ species.

Note that one mouse each did fail in negative and in positive mode, leaving n = 82 samples. Mass spectrometry peak intensities were normalized by the input feces weight and zeros imputed for undetected metabolites. The thus obtained metabolomic feature abundance data was converted to sample wise relative abundance via division with the total of each sample. Each metabolomic feature was then correlated to the mouse age via spearman correlation. Obtained p-values were adjusted for multiple testing via the Benjamini-Hochberg FDR procedure. For plotting in Figure 4F we calculated the log2 fold changes between age 28 and 3 months. Supplementary Table S4.12 summarizes all the identified and age-associated metabolites from spectral library searches and the semi-targeted identification of bile acids.

Aging brain metabolome analysis

To analyze aging-associated changes in the metabolome, data provided by Feng et al. [41] (Supplementary Table S2 in the referenced paper) that comprised metabolites measured across several age groups for ten anatomical brain regions were utilized. Data for three-week-old mice were excluded to avoid confounding by pre-aging trajectories. Metabolites were mapped to the IDs contained in the metamodel using the provided PubChem IDs [116] and information from the BiGG database [117]. Subsequently, for each metabolite, concentrations for each measured tissue were correlated against the age of the mice using Spearman's rank correlation. Only associations with a Benjamini–Hochberg FDR-adjusted [118] p-value of ≤0.05 were retained. In the analysis of microbiome-produced and microbiome-consumed brain metabolites, a metabolite was assumed to be provided from the microbiome to the brain if its number of cases of microbiome-dependent uptake was greater than its number of cases of microbiome-dependent secretion. An uptake or secretion reaction of the brain was assumed microbiome-dependent if its flux range was reduced to ≤10% of its wild-type value when microbiome reactions were blocked.

Analysis of host responses to microbial colonization

We used transcriptomic data of wild-type mice (CONVR), germ-free mice (GF) and germ-free mice conventionalized with fecal material from wild-type mice (CONVD) across five tissues (brain, colon, liver, gonadal white adipose tissue and quadriceps) to elucidate to which extent model-predicted microbiome-dependent reactions were associated with genes differentially active upon microbial colonization.

To predict dependence of host reactions on the microbiome, we used flux variability analysis on each host reaction (with minimal objective function value set to zero) of the metamodel once with all microbiome reactions present and once with all microbiome reactions blocked. If the flux range (i.e. maximal minus minimal flux in a reaction according to flux variability analysis) of a reaction was reduced by at least 50%, we considered a reaction to be microbiome dependent. This was repeated across all metamodels of the individual mice to determine a microbiome dependence frequency for each reaction in each tissue. Reactions were only considered for a tissue if they occurred in at least 40 metamodels. Additionally, we determined an average microbiome dependency frequency for each reaction by averaging across all tissues. The corresponding values can be found in Supplementary Table S3.8.

For each tissue and comparison, differentially expressed genes upon microbial colonization (comparison GF vs. CONVR and GF vs. CONVD) were filtered for significantly upregulated genes (positive log-fold-change and FDR-adjusted p-value ≤0.1), downregulated genes (negative log-fold-change and FDR-adjusted p-value≤0.1) and unregulated genes (FDR-adjusted p-value>0.1). Genes were translated into reactions using the gene-protein-reaction association of the human metabolic model Recon 2.2. Thus, for each set of genes, we obtained all reactions in which at least one of the genes was involved according to model annotation to obtain a list of upregulated reactions, downregulated reactions and unregulated reactions. From the set of unregulated reactions we removed all reactions that also occurred in the list of up- or downregulated reactions. Moreover, we removed all reactions occurring in the list of up- and down-regulated reactions at the same time. Additionally, we determined a set of shared up-,

down and unregulated reactions across tissues as those reactions that occurred in at least three tissues in the corresponding reaction set.

Subsequently, we determined the microbiome dependency of host reactions based on the tissue-specific microbiome dependency frequency predicted by the metamodel. For tissues not covered by the metamodel (gonadal white adipose tissue and quadriceps), we used average microbiome dependency frequencies from all other tissues. For each tissue, we compared microbiome dependency frequency of up-, down- and unregulated host reactions derived from differentially expressed genes from the comparison of GF vs. CONVR and GF vs. CONVD. For each combination, we first used a Kruskal-Wallis test to determine if there are significant group-level differences between the three reaction sets and a subsequent Dunn's test if the Kruskal-Wallis test yielded a p-value≤0.05. Across all combinations and comparisons, Dunn's test p-values were corrected using false-discovery rate control to adjust for multiple testing.

Since genes are often involved in several reactions and reactions associated with several genes, we performed randomization experiments to exclude that our results were due to the structure of gene-protein-reaction associations. To this end, we randomized gene labels in the list of differentially expressed genes, repeated the analysis described above and counted the number of significant differences for each comparison between reaction sets and tissue/condition combination. This was repeated a thousand times. In no randomization run did we encounter the same or a higher number of significant differences like we did in the original analysis (Extended Data Fig. 3A).

Analysis of human serum metabolome associations

To predict microbiome dependence of metabolites, we iteratively added an exchange reaction for each metabolite exchanged via blood in the metamodel and used flux balance analysis to maximize its outflow. Subsequently, we blocked all microbiome reactions and maximized the outflow again. If the outflow without the microbiome was below 50% of the outflow compared to the wild type simulation, a metabolite was assumed microbiome dependent. Determining microbiome dependence across all 52 mice we obtained a microbiome dependency frequency for each metabolite. Subsequently, metabolites were filtered for those that occurred in at least 40 mice. Variance in human serum metabolite concentration explained by microbiome composition was obtained from Bar et al. [33] and mapped via HMDB IDs to the corresponding microbiome dependence of metabolites in blood predicted by the metamodel. We only considered metabolites from Bar et al. for which the statistical model to assess explained variance yielded a significant fit at p<=0.1. The corresponding data can be found in Supplementary Table S3.10.

Supplementary References

107. Grime, J.P. (1977). Evidence for the Existence of Three Primary Strategies in Plants and Its Relevance to Ecological and Evolutionary Theory. Am. Nat. *111*, 1169–1194. https://doi.org/10.1086/283244.

108. Schwengers, O., Jelonek, L., Dieckmann, M.A., Beyvers, S., Blom, J., and Goesmann, A. (2021). Bakta: rapid and standardized annotation of bacterial genomes via alignment-free sequence identification. Microb. Genomics *7*, 000685.

https://doi.org/10.1099/mgen.0.000685.

109. Chen, L., Zheng, D., Liu, B., Yang, J., and Jin, Q. (2016). VFDB 2016: hierarchical and refined dataset for big data analysis—10 years on. Nucleic Acids Res. *44*, D694–D697. https://doi.org/10.1093/nar/gkv1239.

110. Weissman, J.L., Hou, S., and Fuhrman, J.A. (2021). Estimating maximal microbial growth rates from cultures, metagenomes, and single cells via codon usage patterns. Proc. Natl. Acad. Sci. *118*, e2016810118. https://doi.org/10.1073/pnas.2016810118.

111. Diener, C., and Gibbons, S.M. (2023). More is Different: Metabolic Modeling of Diverse Microbial Communities. mSystems *8*, e01270-22. https://doi.org/10.1128/msystems.01270-22.

112. Jantzen, S.G., Sutherland, B.J., Minkley, D.R., and Koop, B.F. (2011). GO Trimming: Systematically reducing redundancy in large Gene Ontology datasets. BMC Res. Notes *4*, 267. https://doi.org/10.1186/1756-0500-4-267.

113. Sillner, N., Walker, A., Lucio, M., Maier, T.V., Bazanella, M., Rychlik, M., Haller, D., and Schmitt-Kopplin, P. (2021). Longitudinal Profiles of Dietary and Microbial Metabolites in Formula- and Breastfed Infants. Front. Mol. Biosci. *8*.

114. Wang, M., Carver, J.J., Phelan, V.V., Sanchez, L.M., Garg, N., Peng, Y., Nguyen, D.D., Watrous, J., Kapono, C.A., Luzzatto-Knaan, T., et al. (2016). Sharing and community curation of mass spectrometry data with GNPS. Nat. Biotechnol. *34*, 828–837. https://doi.org/10.1038/nbt.3597.

115. Sillner, N., Walker, A., Koch, W., Witting, M., and Schmitt-Kopplin, P. (2018). Metformin impacts cecal bile acid profiles in mice. J. Chromatogr. B Analyt. Technol. Biomed. Life. Sci. *1083*, 35–43. https://doi.org/10.1016/j.jchromb.2018.02.029.

116. Kim, S., Chen, J., Cheng, T., Gindulyte, A., He, J., He, S., Li, Q., Shoemaker, B.A., Thiessen, P.A., Yu, B., et al. (2021). PubChem in 2021: new data content and improved web interfaces. Nucleic Acids Res. *49*, D1388–D1395. https://doi.org/10.1093/nar/gkaa971.

117. Norsigian, C.J., Pusarla, N., McConn, J.L., Yurkovich, J.T., Dräger, A., Palsson, B.O., and King, Z. (2020). BiGG Models 2020: multi-strain genome-scale models and expansion across the phylogenetic tree. Nucleic Acids Res. *48*, D402–D406. https://doi.org/10.1093/nar/gkz1054.

118. Benjamini, Y., Drai, D., Elmer, G., Kafkafi, N., and Golani, I. (2001). Controlling the false discovery rate in behavior genetics research. Behav. Brain Res. *125*, 279–284. https://doi.org/10.1016/s0166-4328(01)00297-2.
